# Bawei Chenxiang Wan Ameliorates Cardiac Hypertrophy by Activating AMPK/PPAR-α Signaling Pathway Improving Energy Metabolism

**DOI:** 10.3389/fphar.2021.653901

**Published:** 2021-06-03

**Authors:** Xiaoying Zhang, Zhiying Zhang, Pengxiang Wang, Yiwei Han, Lijun Liu, Jie Li, Yichun Chen, Duxia Liu, Jinying Wang, Xiaoying Tian, Qin Zhao, Fengxia Yan

**Affiliations:** ^1^ Department of Pharmacology, School of Medicine, Xizang Minzu University, Xianyang, China; ^2^ School of Medical Science, Jinan University, Guangzhou, China

**Keywords:** cardiac hypertrophy, energy metabolism, Bawei Chenxiang Wan, isoprenaline, AMPK/PPAR-α

## Abstract

Bawei Chenxiang Wan (BCW), a well-known traditional Chinese Tibetan medicine formula, is effective for the treatment of acute and chronic cardiovascular diseases. In the present study, we investigated the effect of BCW in cardiac hypertrophy and underlying mechanisms. The dose of 0.2, 0.4, and 0.8 g/kg BCW treated cardiac hypertrophy in SD rat model induced by isoprenaline (ISO). Our results showed that BCW (0.4 g/kg) could repress cardiac hypertrophy, indicated by macro morphology, heart weight to body weight ratio (HW/BW), left ventricle heart weight to body weight ratio (LVW/BW), hypertrophy markers, heart function, pathological structure, cross-sectional area (CSA) of myocardial cells, and the myocardial enzymes. Furthermore, we declared the mechanism of BCW anti-hypertrophy effect was associated with activating adenosine 5'-monophosphate (AMP)-activated protein kinase (AMPK)/peroxisome proliferator–activated receptor-α (PPAR-α) signals, which regulate carnitine palmitoyltransferase1β (CPT-1β) and glucose transport-4 (GLUT-4) to ameliorate glycolipid metabolism. Moreover, BCW also elevated mitochondrial DNA-encoded genes of NADH dehydrogenase subunit 1*(ND1*), cytochrome b (*Cytb*), and mitochondrially encoded cytochrome coxidase I (*mt-co1*) expression, which was associated with mitochondria function and oxidative phosphorylation. Subsequently, knocking down AMPK by siRNA significantly can reverse the anti-hypertrophy effect of BCW indicated by hypertrophy markers and cell surface of cardiomyocytes. In conclusion, BCW prevents ISO-induced cardiomyocyte hypertrophy by activating AMPK/PPAR-α to alleviate the disturbance in energy metabolism. Therefore, BCW can be used as an alternative drug for the treatment of cardiac hypertrophy.

## Introduction

Cardiac hypertrophy is initially regarded as a compensatory response of the heart to various physiological or pathological stimuli. However, sustained hypertrophy is maladaptive and induces a transition into functional decompensation, which leads to heart failure and sudden death (Ruwhof and van der Laarse, 2000; Hill and Olson, 2008). Although cardiac hypertrophy is known to occur due to oxidative stress, inflammation, and autophagy, the main cause has not been elucidated (Li et al., 2016; Anthony et al., 2019; Geng et al., 2019). Recently, a number of studies report that metabolic dysfunction is a hallmark of cardiac hypertrophy and heart failure (Tian et al., 2018; Dhyani et al., 2019). Previous findings in our laboratory and others have proved that the cardiac hypertrophy is also characterized by a metabolic switch in the utilization of energy source that uses glucose as an energy source rather than utilizing fatty acid (Ingwall, 2009; Zou et al., 2013; Gao et al., 2015; Yue et al., 2016). The energy metabolism can also contribute to contractile dysfunction and the progressive left ventricular remodeling that are characteristics of the heart failure state (Stanley et al., 2005; Luptak et al., 2018).

AMP-activated protein kinase (AMPK), a heterotrimetric enzyme, plays a further role in the regulation of cardiac cell growth (Xing et al., 2003; Russell et al., 2004) and energy regulation in the hypertrophied heart (Tian et al., 2001). It is activated in response to stresses that lead to an increase in the cellular AMP/ATP ratio, caused either by inhibition of ATP production or by accelerating ATP consumption (Hardie et al., 2012). Studies have demonstrated that activation of AMPK can repress protein synthesis, inhibit glucose phosphorylation and glycolysis, and prevent cardiac hypertrophy (Xing et al., 2003; Proud, 2004; Russell et al., 2004). Peroxisome proliferator–activated receptor-α (PPAR-α), a ligand-activated transcription factor, belongs to the nuclear hormone receptor superfamily which is considered as a critical regulator of myocardial metabolism. PPAR-α is in high levels in tissues such as heart with high energy demands that depend on the oxidation of mitochondrial fatty acids as a primary energy source (Han et al., 2005). Studies have demonstrated that the occurrence of cardiac hypertrophy is associated with the deactivation of PPAR-α (Goikoetxea et al., 2006).

Bawei Chenxiang Wan (BCW) is one of the common Tibetan medicines used for long history of clinical application. As a prescription drug of Tibetan medicine formulas, BCW consists of *Myristica fragrans* (Houtt.), *Aquilaria sinensis* (Lour.) Spreng, *Choerospondias axillaris* (Roxb.) B.L.Burtt & A.W.Hill, *Boswellia carterii* Birdw, *Terminalia chebula* Retz, *Aucklandia costus* Falc, *Bombax ceiba* L, and travertine (Table1). BCW is used to treat acute and chronic cardiovascular and cerebrovascular diseases in clinical practice, which has a protect effect on the heart and cerebral ischemia (Wang et al., 2006; Zhao et al., 2009; Li et al., 2010). In addition, gallic acid (GA), a class of phenolic compounds, is found in *Choerospondias axillaris* (Roxb.) B.L.Burtt & A.W.Hill, *Terminalia chebula* Retz, and *Gossampinus malabarica* (DC.). Some studies demonstrated that GA reduces cardiac hypertrophy, dysfunction, and fibrosis induced by transverse aortic constriction (TAC) stimuli (Jin et al., 2018; Yan et al., 2019). Therefore, whether BCW comprises of GA and others have the effect of anticardiac hypertrophy deserves to be studied.

**TABLE 1:** The composition of Bawei Chenxiang Wan (BCW).

Species	Herbal name	Part of use	Dosage (g)	Ratio (%)
*Myristica fragrans* Houtt*.*	*Myristica fragrans*	Kernel	100	13.3%
*Aquilaria sinensis* (Lour.) Spreng	*Aquilaria sinensis* (Lour.) Gilg	Dry root	100	13.3%
*Choerospondias axillaris* (Roxb.) B.L.Burtt & A.W.Hill	*Choerospondias axillaries* (Rox.) Burtt et Hill.	Fruits	100	13.3%
*Boswellia carterii Birdw*	*Boswellia carterii*	Resin	50	6.7%
*Terminalia chebula* Retz.	*Terminalia chebula* Retz	Fruits	100	13.3%
*Aucklandia costus* Falc.	*Aucklandia lappa* Decne	Fruits	175	23.3
*Bombax ceiba* L	*Gossampinus malabarica* (DC.)Merr.	Dry flower	75	23.3
Travertine	Calcsinter		50	6.7%
Total			750	100%

The concentration/ratio of the eight species plants present in the preparation.

In this article, cardiac hypertrophy model *in vivo* and *in vitro* stimulated by ISO was applied to explore the effect and mechanism of BCW. Our results revealed that BCW played anti-hypertrophy effect by activating AMPK/PPAR-α signaling pathway improving energy metabolism.

## Materials and Methods

### Standardization of Bawei Chenxiang Wan

BCW, originated from “Four-Volume Medical Code,” is a Chinese Tibetan herbal formula, which carried out Tibetan Medicine standards 1995 by Ministry of health of the people’s Republic of China (Table 1). BCW (CAS18017A) used in our experiment was provided by Tibet Ganlu Tibetan Medicine Co., Ltd. (Lhasa, Tibet, P. R. China).

### Chemicals and Antibodies

Isoprenaline (15,267) was obtained from Sigma-Aldrich (St. Louis, MO, United States); anti-GlUT-4(YT5523), anti-AMPK (YT0216), anti-phospho-AMPK (YP0575), anti-PPAR-α (YT 1862), anti-α-Tubulin (YM3217), anti-β-actin (YM3028), and anti-GAPDH (YM3215) were purchased from ImmunoWay Biotechnology Company (TX, United States). Anti-CPT-1β (22170-1-AP) was obtained from proteintech (Wuhan, Hubei, P.R.C). Gallic acid (CAS 149–91-7), protocatechuic acid (CAS 99–50-3), costunolide (CAS 553–21-9), dehydrocostus lactone (CAS 477–43-0), and dehydrodiisoeugenol (CAS 2680–81-1) were purchased from Chengdu Ruifensi Biotechnology Co., Ltd. (Sichuan, P. R. China). Rhodamine phalloidin and 40, 6-diamidino-2-phenylindole (DAPI) were purchased from Invitrogen (Carlsbad, CA, United States).

### Chromatographic Conditions and Instrumentation

A validation HPLC method was applied onto a Shimadzu (Kyoto, Japan) LC-20AT system. Extraction method of test substance: Weigh 2.0 g of sample and put it into volumetric flask, add 25 ml of methanol and 25 ml of water, and ultrasonicate (power 250 W, frequency 33 khz) for 30 min; stand and filter detection conditions: acetonitrile: 0.2% phosphoric acid water, 0–8 min 9% acetonitrile; 8–20 min 9–60% acetonitrile; 20–55 min 60%; 55–56 min 60%–9%; 56–66 min 9% acetonitrile; detection wavelength: 225 nm; flow rate: 1.00 ml/min; and loading volume: 20 μL.

### Cell Culture

The primary culture of neonatal rat cardiomyocytes (NRCMs) was prepared as previously described (Yu et al., 2013). Cells from the hearts of 1–3-day-old Sprague Dawley rats were seeded at a density of 1 × 10^6^ cells per well onto 6-well plates in DMEM supplemented with 10% NBCS and 5-bromodeoxyuridine (0.1 mM).

### Measurement of Cell Surface Area

Cardiomyocytes seeded in 48-well plates were fixed by 4% paraformaldehyde for 10 min at room temperature, followed by 1% Triton-100 treatment for 10 min. After incubation with 0.1% rhodamine phalloidin for 30 min, the cells were washed with phosphate buffer saline and further incubated with DAPI for 10 min.

The images of cell surface area (red) and DAPI (blue) were captured by a high content screening system (ArrayScan VTI; Thermo Fisher Scientific, Rockford, IL, United States), and cell surface area from randomly selected fields (20 for each group) was determined by the built-in image analysis software. Cell surface area was normalized as percentage of DAPI fluorescence intensity.

### RNA Interference

Small interference RNA (siRNA) for AMPK (siAMPK) and negative control siRNA were purchased from GenePharma (Shanghai, China). Cardiomyocytes seeded in 35 mm^2^ dishes were transfected with 200 pmol of the targeted siRNA or negative control siRNA, respectively, using 5 μL lipofectamine 2000 (Invitrogen), according to the manufacturer’s instructions.

### Animals

Animal care and experimental procedures were complied with the Guide for the Care and Use of Laboratory Animals published by the United States National Institutes of Health (NIH Publication No. 85–23, revised 1996) and were approved by the Research Ethics Committee of Xizang Minzu University. Sprague Dawley (SD) rats (male, weighing 180–220 g, SPF grade, certification No. 2015-030) were provided by the Chengdu Dashuo Laboratory Animal Co., Ltd. (Sichuan, China). The total of 60 SD male rats was randomly divided to the following groups (*n* = 10/group): control group, isoprenaline (ISO)-induced cardiac hypertrophy group, ISO + 0.8 g kg^−1^ d^−1^ Bawei Chenxiang wan (BCW), ISO +0.4 g kg^−1^ d^−1^ BCW, ISO +0.2 g kg^−1^ d^−1^ BCW, and ISO + 13.4 mg kg^−1^ d^−1^ captopril.

The rats were administered saline, BCW, or captopril daily by gavage for 5 weeks. Meanwhile, at last week, except the control group, others were subcutaneously (s. c.) injected with isoprenaline (1.5 mg kg^−1^ d^−1^) for 7 consecutive days until the end of the study. After 6 weeks, all rats were anesthetized with 50 mg/kg sodium pentobarbital, then performed using Technos MPX ultrasound system (Vinno, Suzhou, China) (Zhou et al., 2006). Afterward, arterial blood from the abdominal aorta and heart was removed by trimming the left ventricles quickly. For morphometric measures, transverse sections of the heart were fixed with neutral buffered formalin (10%), embedded in paraffin, cut into 5 μm cross sections, and stained with hematoxylin and eosin (H&E).

### Echocardiography

Echocardiographic assessment was performed by a high resolution ultrasound imaging system. The internal dimensions of the left ventricular (LV) cavity, left ventricular posterior wall (LVPW) and left ventricular anterior wall (LVAW) thickness, left ventricular dimension (LVID), heart rate (HR), ejection fraction (EF%), and fractional shortening (FS%) were measured and recorded.

### Creatine Kinase, Lactate Dehydrogenase, and Free fatty acids

The arterial blood samples harvested from all groups were centrifuged at 3,000 rpm for 10 min at 4°C. The plasma supernatant was obtained and stored at −80°C. According to the chemical formula of creatine kinase (CK), catalyzing adenosine triphosphate and creatine to phosphocreatine, ammonium molybdate can be added to react with adenosine triphosphate to produce molybdenum blue to measure the activity of CK.

The dehydrogenase (LDH) was determined by using pyruvic acid and 2, 4 dinitrophenylhydrazine to produce reddish brown pyruvate dinitrophenylhydrazone under alkaline conditions. The levels of lactate dehydrogenase (LD) activity were determined by the method recommended by the Japanese Society of Clinical Chemistry (JSCC) (lactate–pyruvate direction). At 37°C, with NAD as the hydrogen receptor, LDH catalyzed the dehydrogenation of lactate to pyruvate and converted NAD to NADH, where PMS reduced NBT to a violet chromaticity to detect LD.

FFA was determined by the reaction of free fatty acids (FFA) and coenzyme A under the action of acetyl-CoA synthase (ACS), and acetyl-CoA under the action of acetyl-CoA oxidase (ACOD) to generate H_2_O_2_, and then through chromogen under the action of peroxidase (POD) to generate colored substrate. All of these were measured using the respective assay kits (Nanjing jiancheng Bioengineering Institute, Nanjing, China).

### Real-Time Quantitative Polymerase Chain Reaction

According to the manufacturer’s instructions, total RNA was extracted with TRIzol reagent (Takara Biotechnology, Dalian, China). Reverse transcription was performed using the two-step RT kit (Vazmye, China). PCRs were performed with the SYBR Green Quantitative PCR kit using the following primers: atrial natriuretic factor (*ANF*), brain natriuretic peptide (*BNP*), and β-myosin heavy chain (β*-MHC*); *ATP 5*β*, ND1, Cyt b*, and *MT-COI* (listed in Supplementary Table S1) were synthesized by Sangon Biotech Co., Ltd. (Shanghai, China). Quantification was performed using the efficiency-corrected −$2^{\Delta \Delta \text{CT}}$ method with the housekeeping gene GADPH as endogenous control. Data were presented as folding change over the control group.

### Western Blotting

Western blotting assay was performed as previously described (Feng et al., 2015; Li et al., 2018). Equal amount of proteins (30 mg of total proteins) was separated by 8% sodium dodecyl sulfate–polyacrylamide gel electrophoresis (SDS-PAGE), and then transferred onto polyvinylidene fluoride (PVDF) membranes (Millipore). The membranes were incubated with primary antibodies overnight at 4°C, followed by incubating with appropriate horseradish peroxidase–conjugated secondary antibodies at room temperature for 1 h. The blots were developed with super signal chemiluminescent substrate (Diyibio, Shanghai, China) and detected by the ChemiDoc^TM^ XRS+ (Bio-Rad, CA, United States). The intensities of the blots were quantified by Quantity One software and α-Tubulin or GADPH served as a loading control.

### Data Analysis

The statistical analysis was conducted using SPSS 18.0. The data were presented as mean ± SE. Statistical analysis among various groups was performed by one-way analysis of variance (ANOVA) with the Bonferroni *post hoc* test. In all cases, difference between groups was statistically significant when *p* < 0.05.

## Results

### Quality Control Analysis of Bawei Chenxiang Wan Formula

**FIGURE 1:**
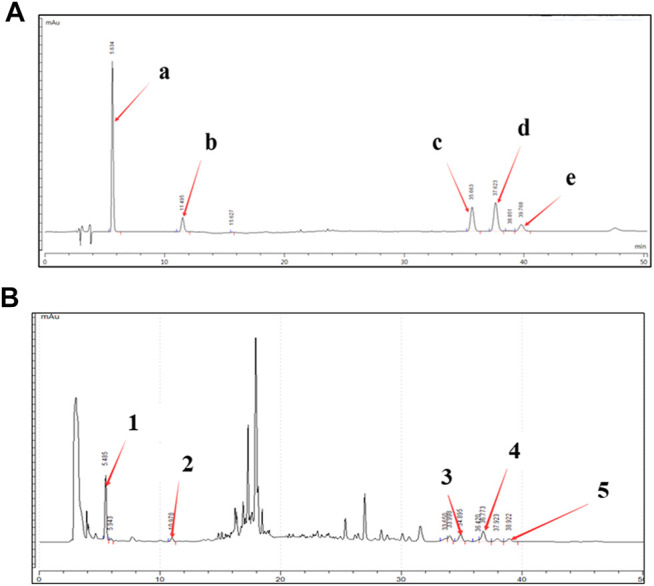
High-performance liquid chromatography (HPLC) chromatogram of the BCW extract. A representative HPLC chromatogram from three batches is shown. **(A)** HPLC chromatograms of mixed reference solutions of five standards: (i) gallic acid, (ii) protocatechuic acid, (iii) costunolide, (iv) dehydrocostus lactone, ( and (v) dehydrodiisoeugenol. **(B)** Five main chemical markers were identified in the BCW extract. The denotation peaks 1–5 were (i) gallic acid, (ii) protocatechuic acid, (iii) costunolide, (iv) dehydrocostus lactone, and (v) dehydrodiisoeugenol.

The high-performance liquid chromatography (HPLC) method was established to reveal the chemical profile of BCW extract and quantify the main ingredients in the extract. The species of the herbs found in BCW are listed in Table 1. Chromatograms of the eight herbs of BCW were analyzed quantitatively (Figure 1A) using a standard curve at 225 nm (Figure 1B), and the calculated concentrations (mg/g) of each compound are summarized in Table 2. We have detected and caculated five main components of BCW, gallic acid (6.5 mg/g), protocatechuic acid (0.2 mg/g), costunolide (2.1 mg/g), dehydrocostusm lactone (2.3 mg/g), and dehydrodiisoeugenol (0.2 mg/g) respectively.

**TABLE 2:** Compounds from BCW.

	Gallic acid	Protocatechuic acid	Costunolide	Dehydrocostus lactone	Dehydrodiisoeugenol
Retention time (min)	5.63	11.56	35.62	37.58	39.73
Content (mg/g)	6.5 ± 0.01	0.2 ± 0.01	2.1 ± 0.01	2.3 ± 0.01	0.2 ± 0.01

Five compounds (gallic acid, protocatechuic acid, costunolide, dehydrocostus lactone, dehydrodiisoeugenol) were identified from BCW, and the minimum amount in mg/g of extract.

### Bawei Chenxiang Wan Treatment Alleviated Isoprenaline-Induced Cardiac Hypertrophy Rats

In the heart, stimulation of β-adrenergic receptors (β-AR) serves as the most powerful means to increase cardiac contractility and relaxation in response to stress. However, sustained β-adrenergic stimulation promotes pathological cardiac remodeling such as myocyte hypertrophy. In our studies, Sprague Dawley rats were subcutaneously injected with 1.5 mg kg^−1^ d^−1^ ISO for 7 days to establish a cardiac hypertrophy model.

**FIGURE 2:**
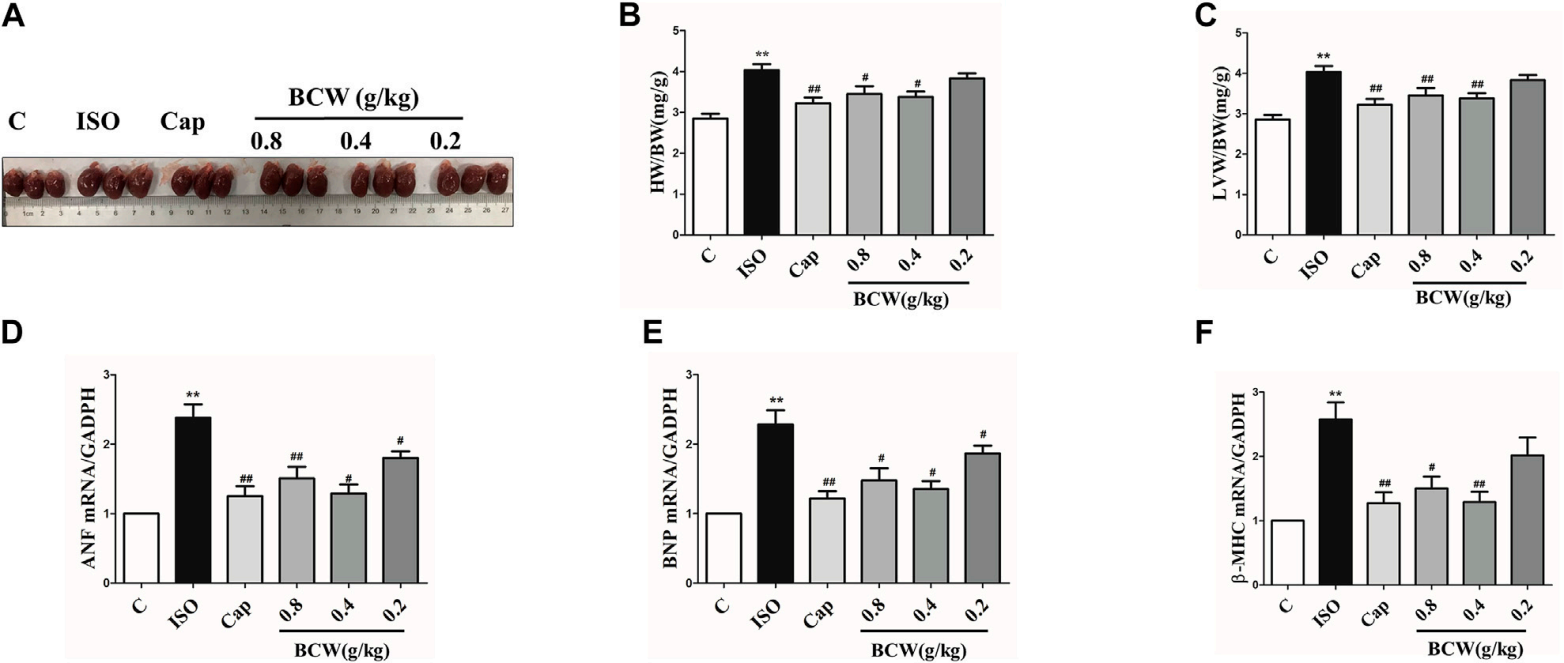
BCW treatment alleviated ISO-induced myocardial hypertrophy. **(A)** Gross hearts from all groups of rats. **(B** and **C)** Quantification of heart weight to body weight ratio and left ventricle heart weight to body weight ratio. **(D** and **E** and **F)** Real-time polymerase chain reaction analysis of the mRNA expression of *ANF, BNP*, and *β-MHC*. Data were normalized by *GADPH* for mRNA expression and presented as mean ± SE, *p < 0.05 vs. control, **p < 0.01 vs. control;^#^ *p* < 0.05 vs. ISO, ^##^ *p* < 0.01 vs. ISO, n = 10.

Hypertrophic indexes were measured in ISO-induced rats to confirm that the model was established. Compared with the control group treated with normal saline, ISO-treated rats demonstrated cardiac hypertrophy, as indicated by the increased heart size, heart weight to body weight ratio (HW/BW), and left ventricle heart weight to bodyweight ratio (LVW/BW). Administration of BCW markedly reduced the heart size, HW/BW, and LVW/BW significantly, especially treatment of BCW with 0.4 g kg^−1^ d^−1^ and 0.8 g kg^−1^ d^−1^ (Figures 2A–C). As cardiac hypertrophy markers, the mRNA expressions of atrial natriuretic factor (*ANF*), brain natriuretic peptide (*BNP*), and β-myosin heavy chain (β*-MHC*) were determined in each group. As shown in Figures 2D,E,F, the expressions of those markers varied in different groups in a fashion similar to that in heart macro morphology, HW/BW, and LVW/BW, suggesting that treatment of BCW 0.4 g kg^−1^ d^−1^and 0.8 g kg^−1^ d^−1^ showed potently beneficial effect in ISO-induced myocardial hypertrophy.

### Bawei Chenxiang Wan Treatment Attenuated Isoprenaline-Induced Heart Dysfunction

**FIGURE 3:**
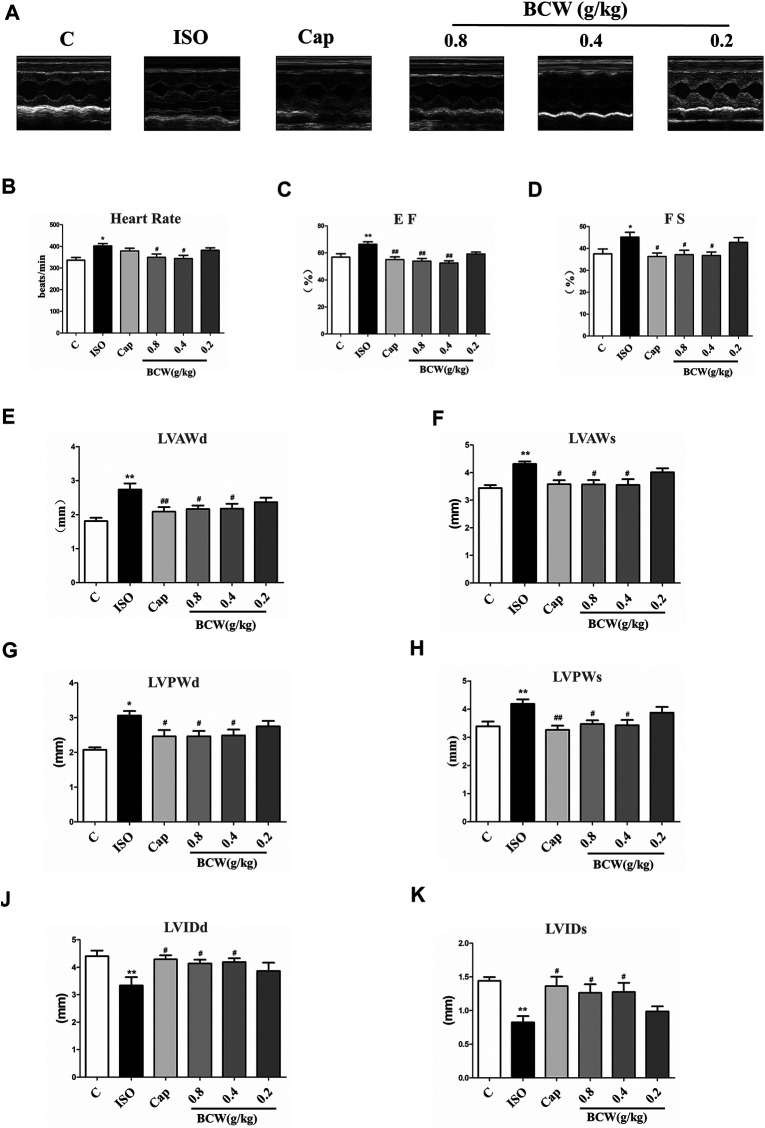
BCW attenuated ISO-induced cardiac functional remodeling. **(A)** Representative M-mode tracings of echocardiography after 5 weeks of the whole experiments. **(B**–**K)** Quantification echocardiographic parameters: **(B)** heart rate (HR); **(C)** ejection fraction **(E**,**F)**; **(D)** fractional shortening (FS); **(E)** left ventricular anterior wall at diastole (LVAWd); **(F)** left ventricular anterior wall at systole (LVAWs); **(G)** left ventricular posterior wall thickness at diastole (LVPWd); **(H)** left ventricular posterior wall thickness at systole (LVPWs) **(I)** left ventricular internal dimension at diastole (LVIDd); and **(J)** left ventricular internal dimension at systole (LVIDds); the data were expressed as the mean ± SE. *p < 0.05 vs. control, **p < 0.01 vs. control; ^#^ *p* < 0.05 vs. ISO, ^##^ *p* < 0.01 vs. ISO, n = 10 in each group.

To further declare the effect of BCW on ISO-induced cardiac functional damage, echocardiography was applied. As shown in Figure 3A, ISO-induced rats showed larger heart size and smaller chamber compared with the control rats, as well as heart rate; EF and FS fractionally increased due to cardiac compensation (Figures 3B–D). Compared with the control group, the heart in theISO group exhibited marked hypertrophy as shown by a significantly increased left ventricular anterior wall thickness at end diastole and systole (LVAWd and LVAWs), as well as LVPWd and LVPWs (left ventricular posterior wall thickness at end diastole and systole) (Figure 3E–H). These alterations were accompanied with decreased left ventricular internal diameter at end diastole and systole (LVIDd and LVIDs) (Figures 3I,J). BCW, especially dose of 0.8 g kg^−1^ d^−1^ and 0.4 g kg^−1^ d^−1^, significantly restored LVPWd, LVPWs, LVAWd, LVAWs, LVIDd, and LVIDs after ISO-treated. In addition, both BCW and captopril treatment decreased the heart rate and EF and FS returned to normal, which were increased by ISO (Figures 3B–D) These results proved that BCW, as well as captopril, prevents the heart against ISO-induced functional deterioration.

### Bawei Chenxiang Wan Suppressed Myocardial Injury Induced by Isoprenaline

**FIGURE 4:**
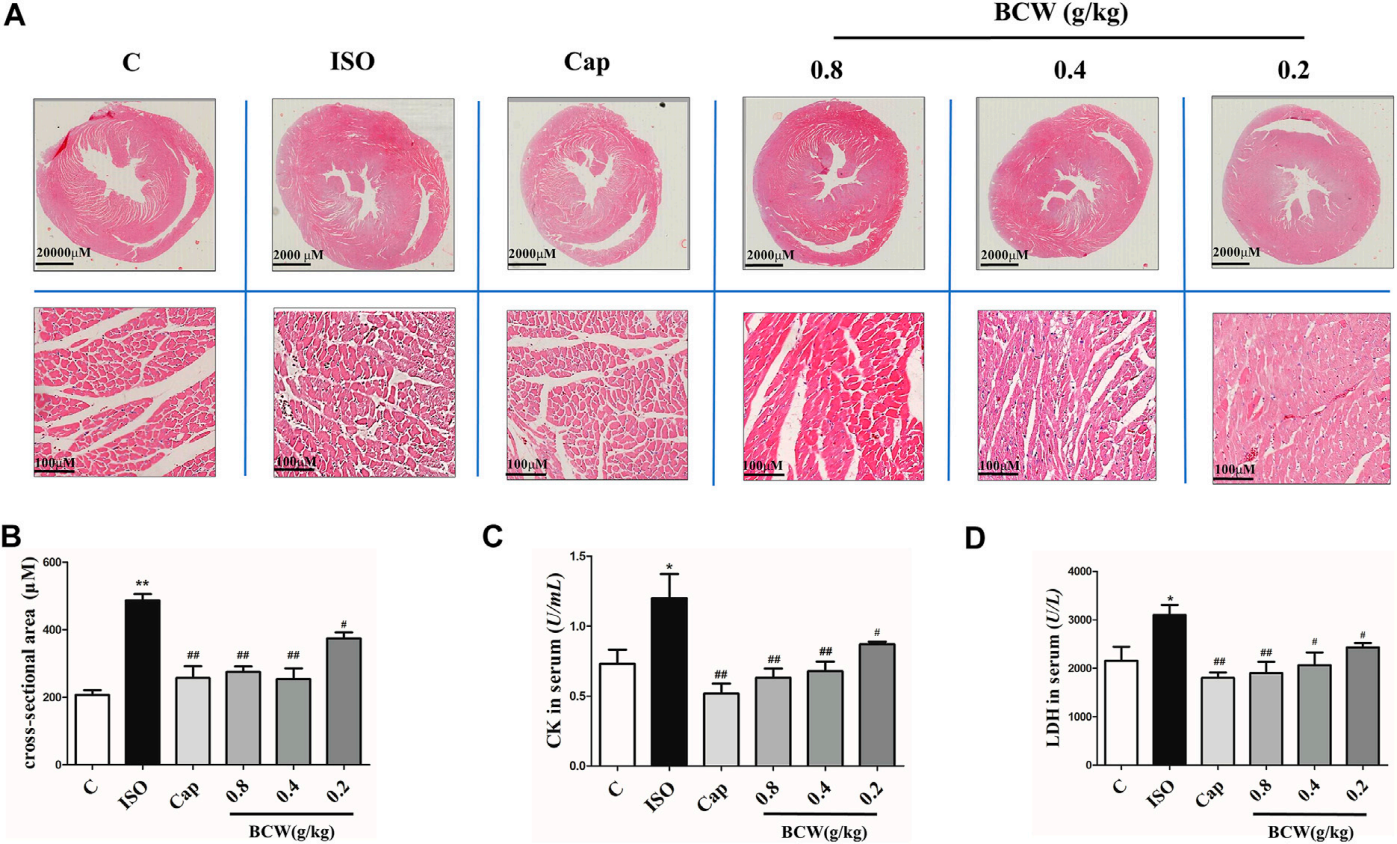
BCW suppressed myocardial hypertrophy induced by ISO. **(A)** Representative slides of cardiac cross sections by hematoxylin and eosin; scale bar in A = 2000 μM, magnification (10×), scale bar in B = 100 μM, magnification (200×). **(B)** cross-sectional area (CSA) of cardiomyocytes, **(C** and **D)** The levels of CK and LDH in serum were measured by commercial kit, respectively. Results are shown as the mean ± SE. *p < 0.05 vs. control, **p < 0.01 vs. control; ^#^ *p* < 0.05 vs. ISO,^##^ *p* < 0.01 vs. ISO, n = 10 in each group.

Rat myocardium from each group was examined by microscopy using hematoxylin and eosin stain (HE). As illustrated in Figure 4A, thickening of ventricular wall, reduction of ventricular volume, and enlargement of cardiomyocyte in the cross section of the heart from the ISO group were observed. Clearly, these ISO-induced morphological alterations in myocardium were attenuated in the rats which received BCW treatment. In Figure 4B, the cross-sectional area (CSA) of cardiomyocytes was increased by induced ISO, but BCW pretreatment was suppressed. Additionally, serum levels of myocardial enzyme were detected by commercial assay kits, respectively. As shown in Figures 4C,D, both lactate dehydrogenase (LDH) and creatine kinase (CK) levels were significantly elevated after ISO but were notably inhibited by BCW, especially in the dose of 0.4 g kg^−1^ d^−1^ and 0.8 g kg^−1^ d^−1^ groups. These investigations indicated that BCW played anti-hypertrophy effect by repressing the size of heart, decreasing hypertrophy markers, improving cardiac function, and alleviating cardiomyocyte injuries.

### Bawei Chenxiang Wan Alleviated Isoprenaline Induced Myocardial Hypertrophy by Modulating Energy Metabolism and Improving Mitochondrial Function

**FIGURE 5:**
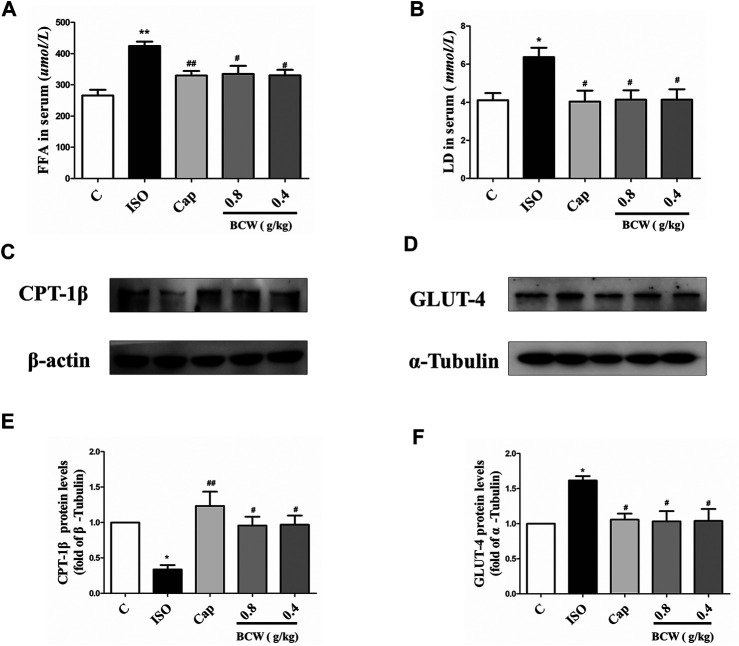
BCW alleviated ISO-induced myocardial hypertrophy by modulating glucolipid metabolism and improving oxidative phosphorylation. **(A** and **B)** The levels of LD and FFA were measured by LD and FFA detection kit, respectively. **(C** and **D)** The protein expression of CPT-1β and GLUT-4 were determined by Western blotting. **(E** and **F)** Densitometric analysis of the immunoblotting was expressed as a percentage of control. Results are shown as the mean ± SE. *p < 0.05 vs. control, **p < 0.01 vs. control; ^#^ *p* < 0.05 vs. ISO,^##^ *p* < 0.01 vs. ISO, n = 10 in each group.

**FIGURE 6:**
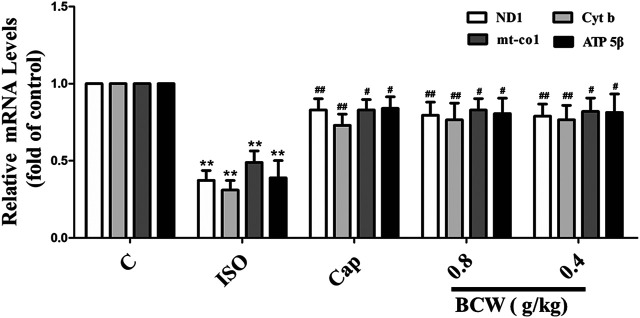
BCW alleviated ISO-induced myocardial hypertrophy by improving mitochondrial function. The mRNA expressions of *ND1, Cytb, mt-co1,* and *ATP 5*β were determined by qRT-PCR. Results are shown as the mean ± SE. *p < 0.05 vs. control, **p < 0.01 vs. control; ^#^ *p* < 0.05 vs. ISO, ^##^ *p* < 0.01 vs. ISO, n = 10 in each group.

We focused on the lactic acid (LD) and free fatty acid (FFA) in the serum to illustrate the glucolipid metabolism underlying the anti-hypertrophy effect of BCW. Consistently, the activation of the β-receptor by ISO forcefully elevated the LD and FFA in serum, which was notably inhibited by BCW (Figures 5A,B). These data showed that the metabolic changes occurred in cardiac hypertrophy, in which glycolysis increased and fatty acid oxidation rates decreased. Furthermore, a series of key enzymes and genes with glycolipid metabolism, oxidative phosphorylation, and mitochondrial function was detected by Western blotting and real-time PCR. As is presented in Figures 5C–F, the protein levels of CPT-1β decreased, GLUT-4 increased by ISO, while BCW significantly reversed these changes. Moreover, the mRNA of mitochondrial DNA-encoded genes including *ND1*, *Cyt b*, and *mt-coI* was diminished treated by ISO, which were increased by BCW, especially in the dose of 0.8 g kg^−1^ d^−1^ and 0.4 g kg^−1^ d^−1^ groups (Figure 6). These data implied that the application of BCW not only increased utilization of free fatty acids and decreased transport of glucose and glycolysis but also improved mitochondrial function.

### Bawei Chenxiang Wan Repressed Isoprenaline-Induced Cardiac Hypertrophy by Up-Regulation of Adenosine 5'-Monophosphate–Activated Protein Kinase/Peroxisome Proliferator–Activated Receptorα Signaling Cascade

**FIGURE 7:**
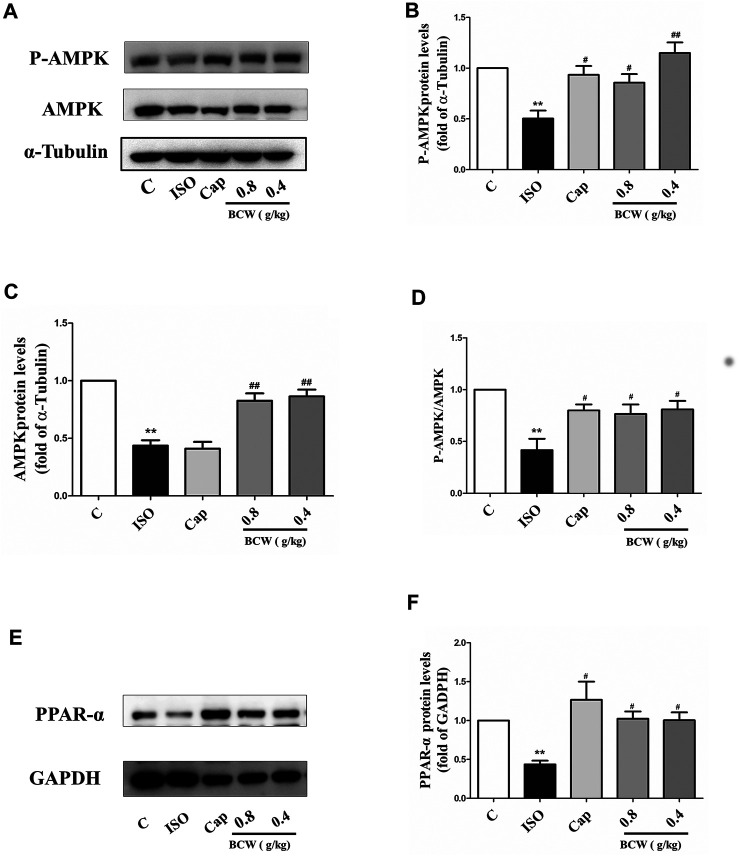
BCW suppressed ISO-induced myocardial hypertrophy by upregulated adenosine 5'-monophosphate–activated protein kinase/peroxisome proliferator–activated receptor-α (AMPK/PPAR-α) signaling cascade. **(A)** The protein expression of total AMPK and phosphorylated P-AMPK was determined by Western blotting. **(B**–**D)** Densitometric analysis of the immunoblot was expressed as a percentage of control. **(E)** Western blot analysis was conducted to determine the protein expression of PPAR-α. **(F)** Densitometric analysis of the immunoblot was expressed as a percentage of control. Data were normalized by α-Tubulin or GADPH for protein expression and presented as mean ± SE. *p < 0.05 vs. control, **p < 0.01 vs. control; ^#^ *p* < 0.05 vs. ISO,^##^ *p* < 0.01 vs. ISO, n = 10 in each group.

To further illustrate the anticardiac hypertrophy effect and the mechanism of BCW, the protein levels of phosphorylation of AMPK, total AMPK, and PPAR-α were explored by Western blotting. As shown in the Figure 7, phosphorylation of AMPK, total AMPK, and P-AMPK/AMPK ratio, as well as PPAR-α, was significantly decreased in response to ISO, which was reversed by BCW remarkably (Figures 7A–F). These data suggested that BCW alleviated ISO-induced myocardial hypertrophy *via* upregulated AMPK/PPAR-α signaling pathway and subsequently improved energy metabolism disorder.

### Adenosine 5'-Monophosphate–Activated Protein Kinase Silencing Reversed the Anti-Hypertrophy Effect of Bawei Chenxiang Wan in Neonatal Rat Cardiomyocytes Induced by Isoprenaline

**FIGURE 8:**
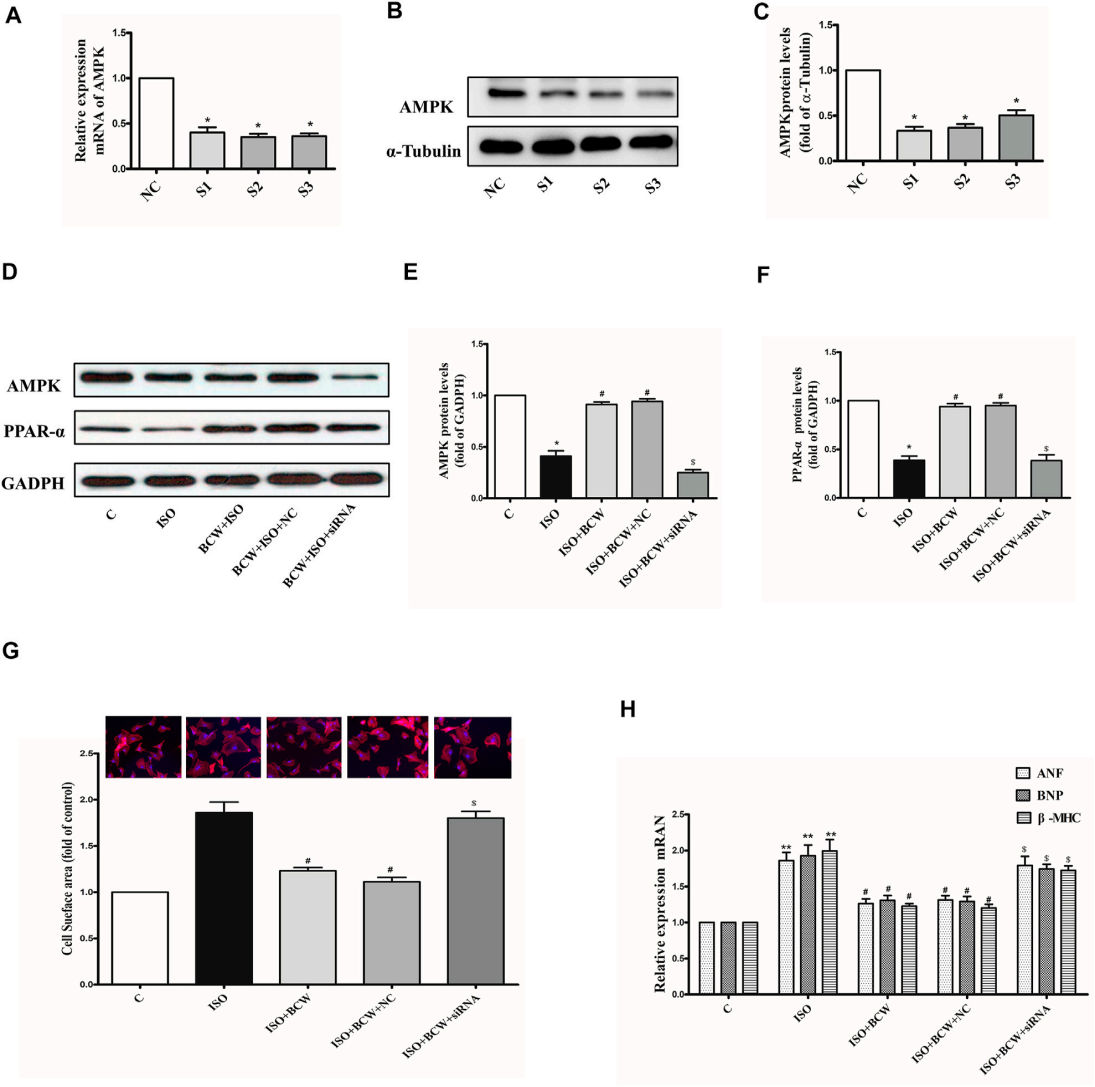
Adenosine 5'-monophosphate–activated protein kinase (AMPK) silencing the reversed anti-hypertrophy effect of BCW in neonatal rat cardiomyocytes induced by ISO. **(A)** mRNA and **(B**–**C)** protein expression of AMPK in cardiomyocytes transfected with three different siRNAs for AMPK or negative control (NC) for 48 h. **(D**–**F)** Protein expression of AMPK and PPAR-α in primary neonatal rat cardiomyocytes infected with or without AMPK siRNA or the NC for 48 h, or treated with or without 10 μM ISO for 24 h. **(G)** Cell surface area measured by rhodamine phalloidin staining (red for rhodamine phalloidin and blue for DAPI) and in primary neonatal rat cardiomyocytes infected with or without AMPK siRNA or the NC for 48 h, or treated with or without 10 μM ISO for 24 h (observed at 200× magnification). **(H)** The mRNA expressions of *the ANF, BNP*, and β*-MHC* determined by qRT-PCR in primary neonatal rat cardiomyocytes infected with or without AMPK siRNA or the NC for 48 h, or treated with or without 10 μM ISO for 24 h (observed at 200× magnification). Data were normalized by GADPH for mRNA expression and protein expression and presented as mean ± SE. *p < 0.05 vs. control; ^#^ *p* < 0.05 vs. ISO, ^$^ *p* < 0.05 vs. ISO + BCW + NC *n* = 3.

RNA interference was used to knock down the endogenous AMPK in the neonatal rat cardiomyocytes (NRCMs) to determine the mechanism of BCW anti-hypertrophy effect. In Figures 8A–C, the mRNA and protein expression levels of AMPK were significantly reduced by siRNA-S3. ISO, an a-beta receptor agonist, induces cardiomyocyte hypertrophy. Our observations revealed that 10 μM ISO administered for 24 h significantly increased the mRNA levels of ANF, BNP, and β *-MHC* in the NRCMs (Figure 8H), and similar results were observed in the cell surface of cardiomyocytes. This result suggested that the ISO-induced hypertrophic cardiomyocyte model was successfully established. Meanwhile, the protein expression of AMPK and PPAR-α was decreased in response to ISO. As shown in the Figure 8, treatment with BCW (100 mg/l) not only activated the expression of AMPK and PPAR-α but also alleviated cardiomyocyte hypertrophy indicated by hypertrophy markers and cell surface of cardiomyocytes. However, knock down of AMPK significantly reversed the effect of BCW. These data suggested that BCW attenuated cardiac hypertrophy by inhibition of AMPK/PPAR-α partly.

## Discussion

This study revealed the protection of BCW on ISO-induced cardiac hypertrophy *in vivo* and *in vitro*, as showed by improved heart function, macro and micromorphology, and cardiac hypertrophy markers, which were associated with improving the energy metabolism by activating the AMPK/PPAR-α signaling pathway. All these evidences showed that BCW may be a potential option for protection of cardiac hypertrophy.

Cardiac hypertrophy is characterized by reactivation of the fetal gene program, increase in protein synthesis, and subsequent increase in the size of the cardiomyocytes (Rohini et al., 2010; Zhang et al., 2016; Prathapan et al., 2017). In our study, β-adrenoreceptor agonist ISO by subcutaneous administration was established to cardiac hypertrophy model *in vivo and in vitro*. The mRNA levels of hypertrophy marker ANF, BNP, β-MHC, cell surface of cardiomyocytes, and size of the cardiomyocytes, as well as the HW/BW ratio and LVW/BW ratio, were elevated after being ISO-treated similar to earlier reports (Feng et al., 2015; Jernberg et al., 2017) (Figures 2, 4A, 8D,E).

More importantly, the ISO stimulates cardiac adrenergic receptors directly, which result in intracellular calcium overload that leads to myocardial contraction excessively (Liles et al., 2011). In this study, ISO-induced excessive myocardial contraction resulted in left ventricular functional impairment, as indicated by increased EF and FS (Figures 3C,D). Meanwhile, LVAWd, LVAWs, LVPWd, and LVPWs (indicator of ventricular wall thickness) increased and the LVIDds and LVIDs (indicator of ventricular volume) decreased (Figures 3E–K). Furthermore, the CK system plays an important role in myocardial energy metabolism (Tian et al., 2018). ISO damages the myocardial cells, which contain LDH, CK-MB, and AST, resulting in leakage of enzymes in the blood (Ghule et al., 2009). Other studies showed that increased activities of CK-MB, LDH, and SGOT in hypertrophied rats indicate myocyte death, early energy imbalance, and inadequate myocardial adaptation (A et al., 2017). Similar results have been found in our study, in which the CK and LDH in serum were elevated significantly in hypertrophied rats (Figures 4B,C).

A number of studies report that Bawei Chenxiang powder has a protective effect on myocardial ischemia (Wang et al., 2006; Zhao et al., 2009; Li et al., 2010). The mechanism includes antioxidant, anti-apoptosis, improving cardiac systolic and diastolic function, and protecting the structure of mitochondrion, which are positive against myocardial hypertrophy. Moreover, GA, as a main content in BCW, might be a novel therapeutic agent for the prevention of cardiac hypertrophy and fibrosis by regulating the JNK2 and Smad3 signaling pathway (Ryu et al., 2016). Hence, we discuss the effect and mechanism of BCW for myocardial hypertrophy. Our study found that BCW treatment not only decreased the size of the heart, hypertrophy markers, cell surface of cardiomyocytes but alleviated the histopathological changes and impairment of cardiomyocytes (Figures 2, 4, 8). Furthermore, BCW also improved cardiac systolic and diastolic function by decreasing the ventricular wall thickness while increasing volume, which is consistent with previous studies (Yang et al., 2011) (Figure 3). Taken together, BCW can protect ISO-induced cardiac hypertrophy.

The heart has more energy demand because of its continuous contractile activity. An altered utilization of metabolic substrates is recognized as one of the biochemical hallmarks of the cardiac hypertrophy and failing heart (Kumar et al., 2019). A number of studies indicated that cardiac energy metabolism occurred unavoidably in cardiac hypertrophy rat models induced by ISO or AAC (Gao et al., 2015; Dhyani et al., 2019). CPT-1 mediates the uptake of long-chain fatty acids into mitochondria and plays an important role in the control and regulation of mitochondrial β-oxidation (Jernberg et al., 2017). The expression of CPT-1β decreased in the ISO group, which indicated the utilization of fatty acids is hindered, resulting in FFA being increased (Figures 5A,C,E). Moreover, the increase in LD and glycolysis products could potentially be due to enhanced GLUT-4 into hypertrophy myocytes induced by ISO (Figures 5B,D,F). More importantly, BCW treatment alleviated disturbance of glucose and lipid metabolism by upregulating CPT-1β and downregulating GLUT-4, which resulted in both FFA and LD being decreased.

Mitochondria produces adenosine triphosphate (ATP) for energy requirements *via* the mitochondrial oxidative phosphorylation (OXPHOS) system, which consists of four protein complexes to generate the proton gradient over the inner and outer membrane of mitochondria and complex V to utilize the proton gradient to produce ATP (Fernandez-Vizarra et al., 2009). Generally, structural abnormalities inevitably lead to functional disorders. Moreover, the mRNA of *ND1*, *Cyt b*, *and mt-coI*, *as well as ATP 5*β, was decreased in ISO group, whereas BCW could reverse (Figure 6). Accumulating evidences demonstrate that BCW alleviates ISO-induced cardiac hypertrophy in rats at least partly by ameliorating dysregulation of energy metabolism and mitochondrial function.

Subsequently, to explore the mechanisms of BCW anti-hypertrophy effect, we detected some proteins with energy metabolism. AMPK, as a sensor of metabolic stress, regulated cardiac glucose metabolism and energy homeostasis, which has a protective effect on the development of critical pathologies like diabetes, myocardial ischemia, and cardiac hypertrophy (Daskalopoulos et al., 2016; Chen et al., 2019a; Chen et al., 2019b)

Mutations in γ^2^ subunits of AMPK not only cause glycogen overload in the heart but also hypertrophy and heart failure (Gollob et al., 2001a; Gollob et al., 2001b). Studies have demonstrated that the activation of AMPK inhibits protein synthesis and the development of cardiac hypertrophy *via* a number of pathways, such as eukaryotic elongation factor-2 (eEF2), p70S6 kinase, and mammalian target of rapamycin (mTOR) (Proud, 2004) AICAR, as an agonist of AMPK, can activate phosphorylation of AMPK-α catalytic subunits (Thr172) to prevent cardiac hypertrophy induced by phenylephrine (Huang et al., 2014). Besides, metformin and oligomycin activated AMPK and inhibited glucose phosphorylation and glycolysis in rat hepatocytes (Meng et al., 2011). In our experiments, BCW can upregulate AMPK *in vivo and in vitro* cardiac hypertrophy model (Figures 7A, 8A). Therefore, we deduce this may be the main mechanism of BCW in the anti-hypertrophy effect.

PPAR-α, which is downstream effector of AMPK, thereby regulates glucose uptake and fatty acid synthesis and oxidation. The previous study declared that fenofibrate ascended the fatty acid β-oxidation enzymes by activating PPAR-α, promoting fatty acid oxidation in the mitochondria, and ameliorating myocardial energy metabolism (Li et al., 2015). Furthermore, CPT-1 is the down target gene of PPAR-α (Toral et al., 2015). Our results also showed that both PPAR-α and CPT-1 expression decreased in the ISO group. However, the alters were reversed by BCW (Figures 5C,E, 7E,F).

The most interesting finding of the present study is disclosure of the role of BCW in activating AMPK to prevent cardiomyocyte hypertrophy. In ISO-induced cardiomyocyte hypertrophy model *in vivo* and in *vitro*, BCW treated can upregulate AMPK and PPAR-α, then inhibit hypertrophy marker and cell surface of cardiomyocytes (Figures 2, 7, 8D–H–).While AMPK is knocked down by siRNA, the anti-hypertrophy effect of BCW decreased (Figures 8D,E). Therefore, we conclude that BCW prevents ISO-induced cardiomyocyte hypertrophy *via* activating AMPK/PPAR-α to alleviate the disturbance in energy metabolism partly.

In this article, we detected five compounds (gallic acid, protocatechuic acid, costunolide, dehydrocostus lactone, and dehydrodiisoeugenol) derived from BCW in HPLC (Table 2). The polyphenolic compound gallic acid (GA) plays its role through the activation of AMP-activated protein kinase (AMPK) and by regulating mitochondrial function *via* the activation of peroxisome proliferator–activated receptor-γ coactivator1α (PGC1α), the interscapular brown adipose tissue genes related to thermogenesis (Guigas et al., 2006). The administration of GA markedly improves cardiac dysfunction and fibrosis in a mouse model of pressure overload–induced cardiac myocyte hypertrophy and heart failure in primary rat cardiac fibroblasts (Jin et al., 2018; Yan et al., 2019). However, no studies have found that the other four components have anticardiac hypertrophy effect. But we will keep the interest of these and probably conduct the study in the future.

## Conclusion

In summary, our results demonstrated that BCW can repress cardiac hypertrophy induced by ISO through improving energy metabolism disturbance. Besides, BCW protected against cardiac hypertrophy by activating the AMPK/PPAR-α pathway and ameliorating myocardial energy metabolism. These findings provide notable support for BCW protection for cardiac hypertrophy.

## Data Availability

The raw data supporting the conclusions of this article will be made available by the authors, without undue reservation, to any qualified researcher.

## Abbreviations

ANF, atrial natriuretic factor; BCW, Bawei Chenxiang Wan; BNP, brain natriuretic peptide; β–MHC, β-myosin heavy chain; CK, creatine kinase; CPT-1β, Carnitine palmitoyltransferase 1β; Cyt b, cytochrome b; FFA, free fatty acid; GLUT-4, glucose transport-4; HE, hematoxylin-eosin; HW/BW, heart weight to body weight ratio; ISO, Isoprenaline; LDH, lactate dehydrogenase; LD, lactic acid; LVAW, left ventricular anterior wall thickness; LVID, left ventricular internal diameter; LVPW, left ventricular posterior wall thickness; LVW/BW, left ventricle heart weight to body weight ratio; mt-co1, mitochondrially encoded cytochrome coxidase I; ND1, NADH dehydrogenase subunit 1.
