# Supplementary material for: Bawei Chenxiang Wan Ameliorates Cardiac Hypertrophy by Activating AMPK/PPAR-α Signaling Pathway Improving Energy Metabolism
Source: Front Pharmacol. 2021 Jun 3;12:653901. doi: 10.3389/fphar.2021.653901 (PMC8209424; doi:10.3389/fphar.2021.653901)
Supplement: Supplementary file 1 [file DataSheet1.ZIP › 653901/Figure5 and Figure7.pptx]

## Slide 1
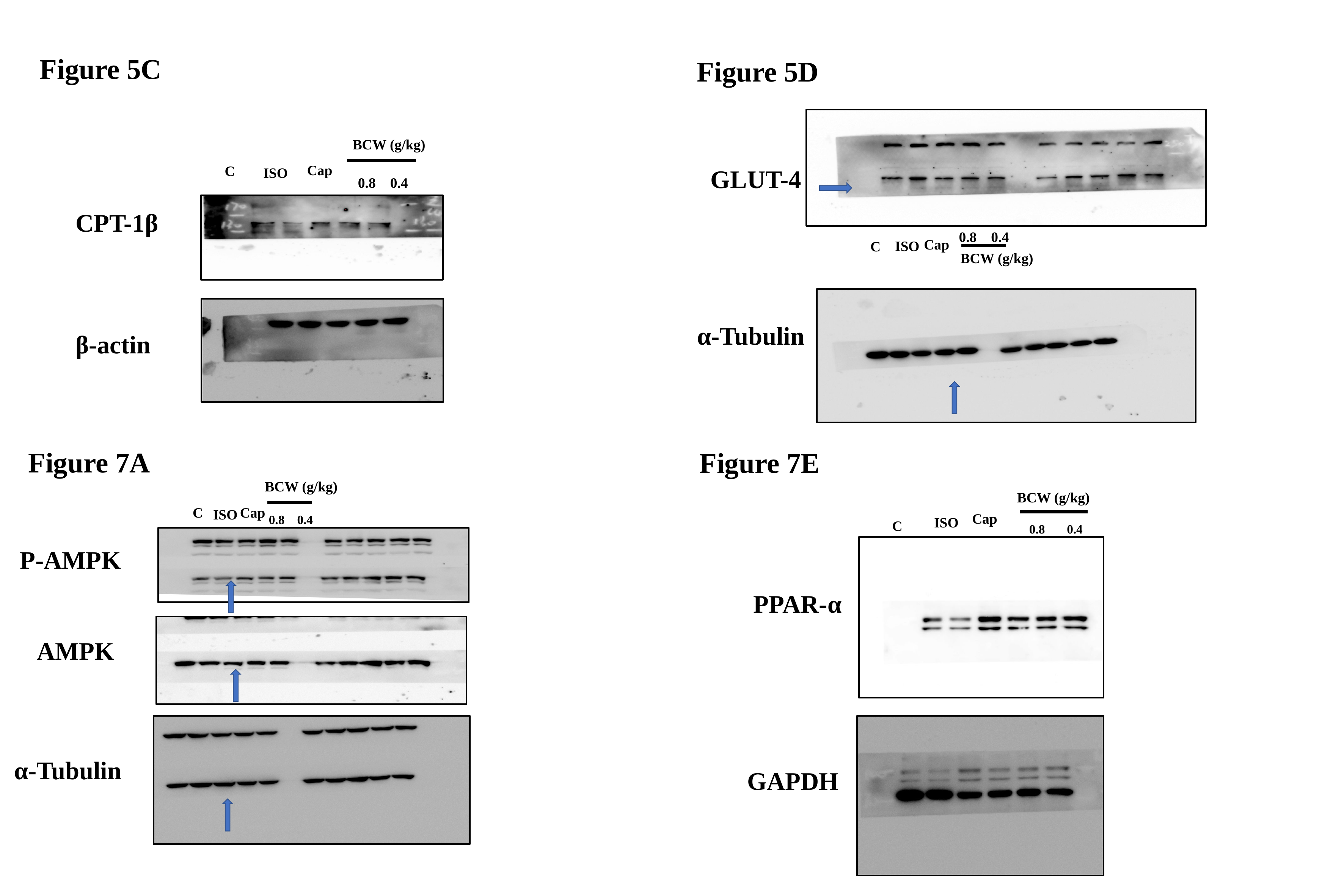

Figure 5C
Figure 5D
BCW (g/kg)
Cap
C
GLUT-4
ISO
 0.8 0.4
CPT-1β
 0.8 0.4
Cap
ISO
C
BCW (g/kg)
α-Tubulin
β-actin
Figure 7A
Figure 7E
BCW (g/kg)
BCW (g/kg)
C
Cap
ISO
Cap
 0.8 0.4
ISO
C
 0.8 0.4
P-AMPK
PPAR-α
AMPK
α-Tubulin
GAPDH
