# Supplementary material for: Bawei Chenxiang Wan Ameliorates Cardiac Hypertrophy by Activating AMPK/PPAR-α Signaling Pathway Improving Energy Metabolism
Source: Front Pharmacol. 2021 Jun 3;12:653901. doi: 10.3389/fphar.2021.653901 (PMC8209424; doi:10.3389/fphar.2021.653901)
Supplement: Supplementary file 1 [file DataSheet1.ZIP › 653901/Figure8.pptx]

## Slide 1
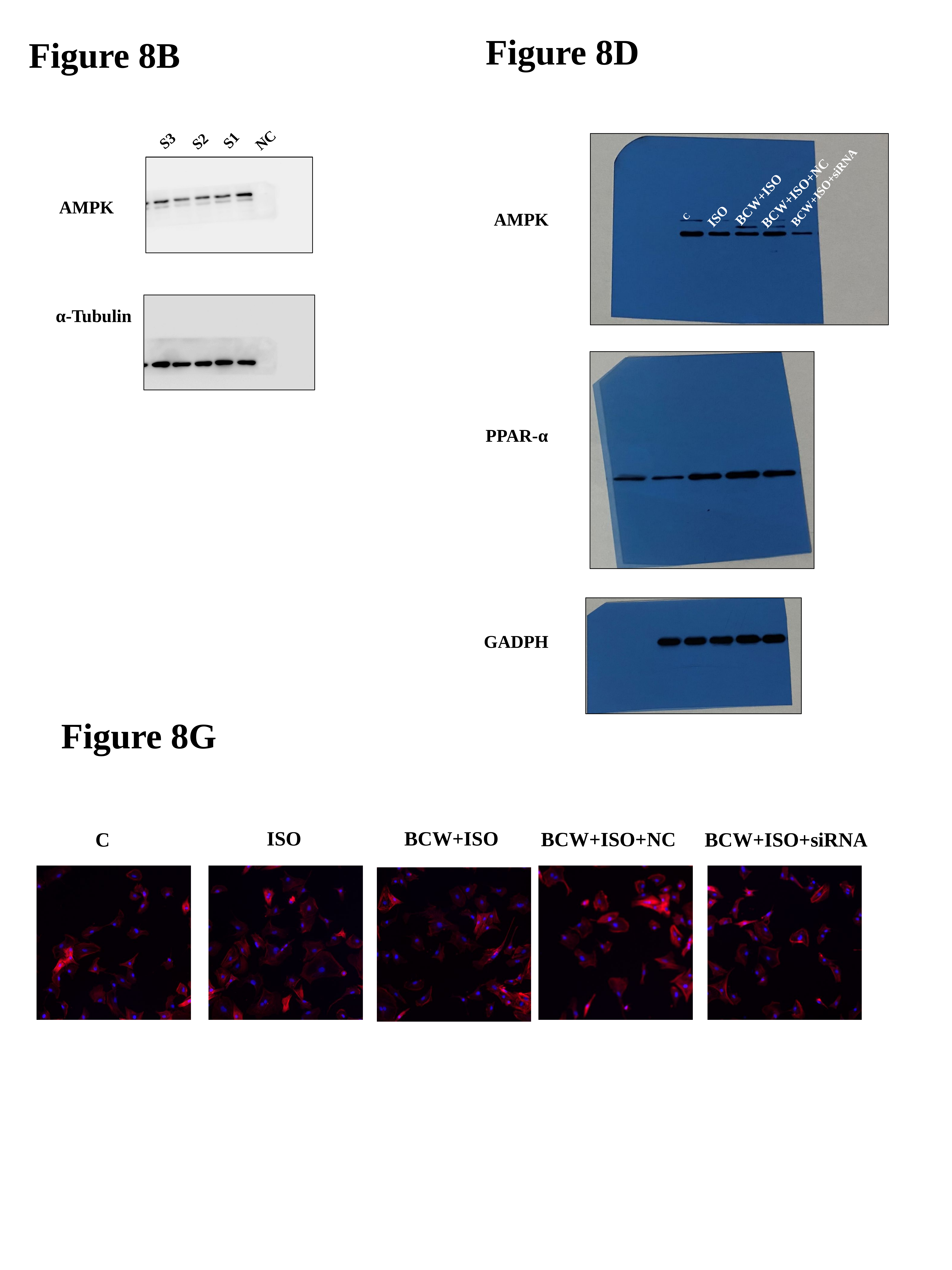

Figure 8D
Figure 8B
S2
NC
S1
S3
BCW+ISO+siRNA
BCW+ISO+NC
ISO
BCW+ISO
AMPK
AMPK
C
α-Tubulin
PPAR-α
GADPH
Figure 8G
ISO
BCW+ISO
BCW+ISO+NC
C
BCW+ISO+siRNA
