# Supplementary figures and images for: Bawei Chenxiang Wan Ameliorates Cardiac Hypertrophy by Activating AMPK/PPAR-α Signaling Pathway Improving Energy Metabolism
Source: Front Pharmacol. 2021 Jun 3;12:653901. doi: 10.3389/fphar.2021.653901 (PMC8209424; doi:10.3389/fphar.2021.653901)

## Slide 1
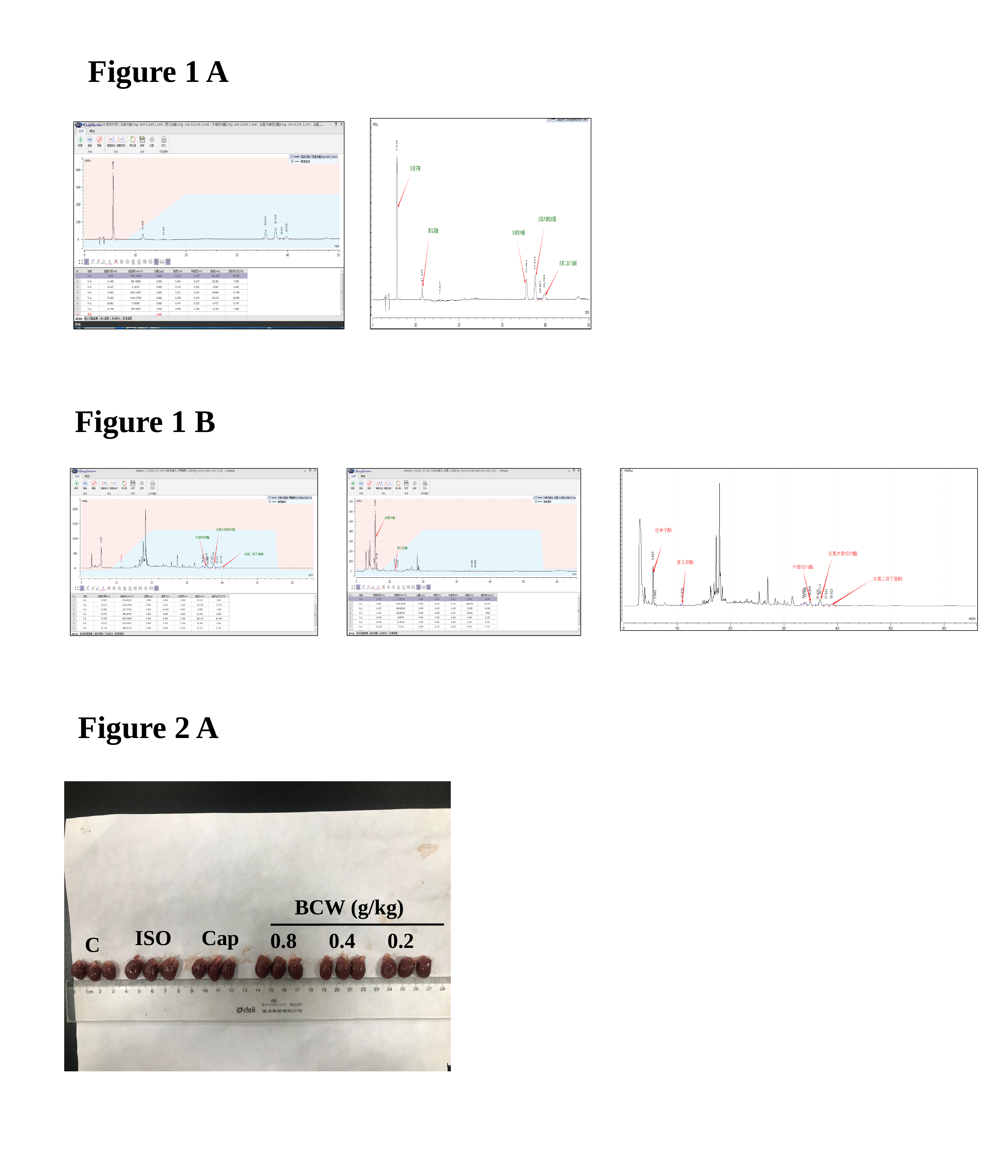

Figure 1 A
Figure 1 B
Figure 2 A
BCW (g/kg)
ISO
Cap
 0.8 0.4 0.2
C

Supplement: Supplementary file 1 [file DataSheet1.ZIP › 653901/Figure1 and Figure2.pptx]

## Slide 1
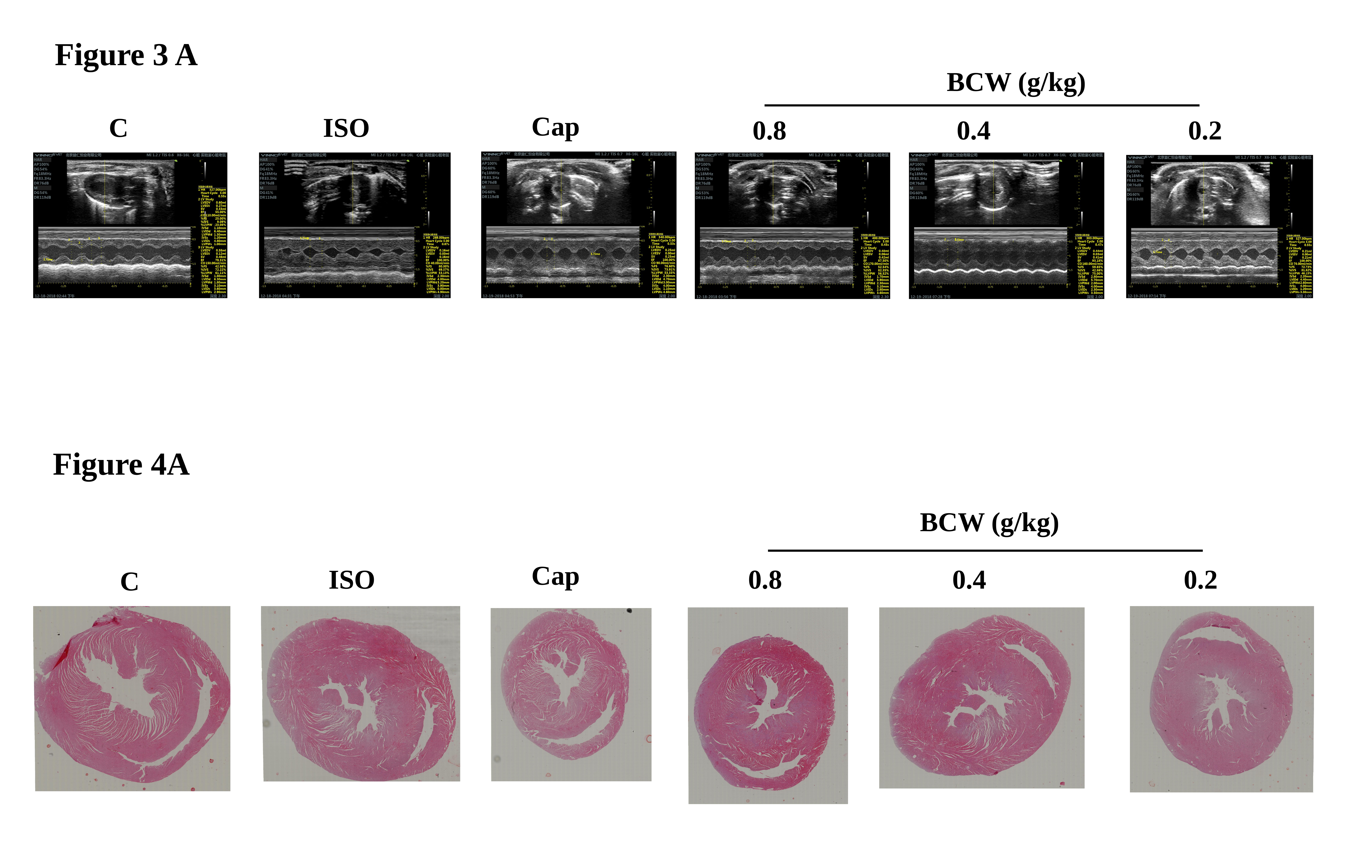

Figure 3 A
BCW (g/kg)
Cap
ISO
C
 0.8 0.4 0.2
Figure 4A
BCW (g/kg)
Cap
ISO
 0.8 0.4 0.2
C

Supplement: Supplementary file 1 [file DataSheet1.ZIP › 653901/Figure3 and Figure4.pptx]
